# Methyl 2-{2-[(2-methyl­phen­oxy)meth­yl]phen­yl}-2-oxoacetate

**DOI:** 10.1107/S1600536813008878

**Published:** 2013-04-10

**Authors:** Manpreet Kaur, Ray J. Butcher, Jerry P. Jasinski, H. S. Yathirajan, B. P. Siddaraju

**Affiliations:** a Department of Studies in Chemistry, University of Mysore, Manasagangotri, Mysore 570 006, India; b Department of Chemistry, Howard University, 525 College Street NW, Washington, DC 20059, USA; c Department of Chemistry, Keene State College, 229 Main Street, Keene, NH 03435-2001, USA; d Department of Chemistry, G. Madegowda Institute of Technology (GMIT), Bharathinagara 571 422, India

## Abstract

In the title compound, C_17_H_16_O_4_, the dihedral angle between the benzene rings is 4.4 (2)°. In the crystal, weak C—H⋯O hydrogen bonds connect mol­ecules along [001].

## Related literature

The title compound is used in organic synthesis as a fungicide inter­mediate. For background to agrochemical fungicidal activity, see: Balba (2007 ▶); Cash & Cronan (2001 ▶); Ammermann *et al.* (2000 ▶); For related structures see: Chopra *et al.* (2004 ▶); Kant *et al.* (2012 ▶). For standard bond lengths, see: Allen *et al.* (1987 ▶).
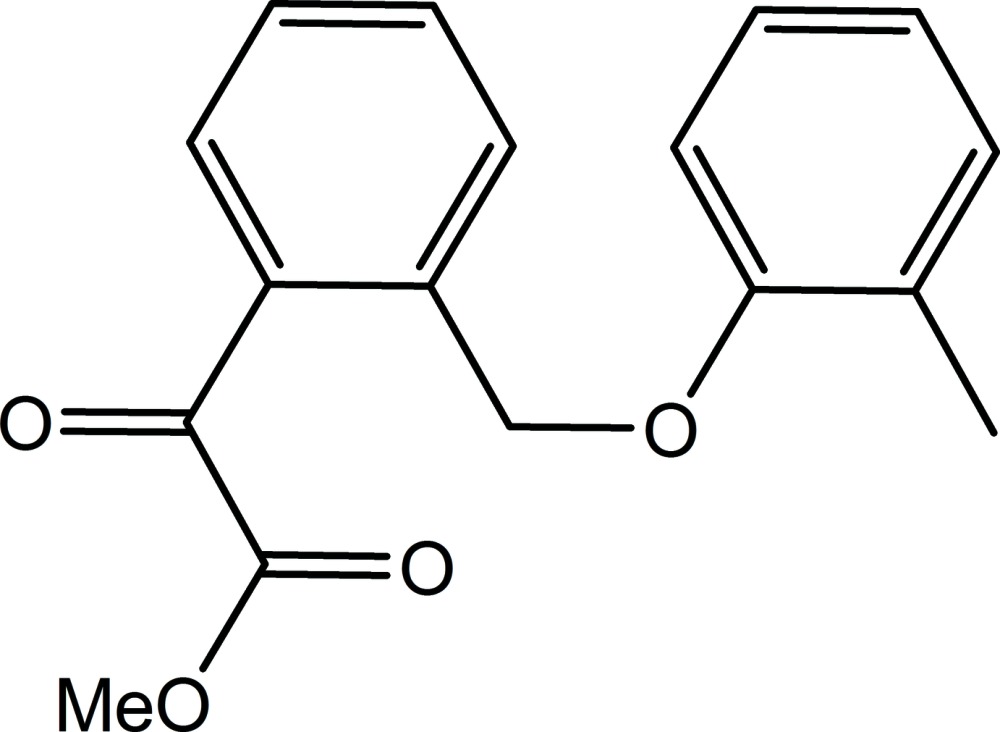


## Experimental

### 

#### Crystal data

C_17_H_16_O_4_
*M* *_r_* = 284.30
Monoclinic, 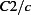

*a* = 31.6697 (11) Å
*b* = 7.5883 (2) Å
*c* = 12.5915 (6) Å
β = 108.514 (4)°
*V* = 2869.4 (2) Å^3^
*Z* = 8
Cu *K*α radiation
μ = 0.77 mm^−1^
*T* = 123 K
0.47 × 0.34 × 0.14 mm

#### Data collection

Agilent Xcalibur (Ruby, Gemini) diffractometer
Absorption correction: multi-scan (*CrysAlis RED*; Agilent, 2012 ▶) *T*_min_ = 0.921, *T*_max_ = 1.000
5506 measured reflections
2897 independent reflections
2551 reflections with *I* > 2σ(*I*)
*R*_int_ = 0.021

#### Refinement

*R*[*F* ^2^ > 2σ(*F* ^2^)] = 0.038
*wR*(*F* ^2^) = 0.107
*S* = 1.07
2897 reflections
192 parameters
H-atom parameters constrained
Δρ_max_ = 0.23 e Å^−3^
Δρ_min_ = −0.20 e Å^−3^

### 

Data collection: *CrysAlis PRO* (Agilent, 2012 ▶); cell refinement: *CrysAlis PRO*; data reduction: *CrysAlis PRO*; program(s) used to solve structure: *SHELXS97* (Sheldrick, 2008 ▶); program(s) used to refine structure: *SHELXL2012* (Sheldrick, 2008 ▶); molecular graphics: *OLEX2* (Dolomanov *et al.*, 2009 ▶); software used to prepare material for publication: *OLEX2*.

## Supplementary Material

Crystal structure: contains datablock(s) global, I. DOI: 10.1107/S1600536813008878/lh5601sup1.cif

Structure factors: contains datablock(s) I. DOI: 10.1107/S1600536813008878/lh5601Isup2.hkl

Supplementary material file. DOI: 10.1107/S1600536813008878/lh5601Isup3.cml

Additional supplementary materials:  crystallographic information; 3D view; checkCIF report

## Figures and Tables

**Table 1:** Hydrogen-bond geometry (Å, °)

*D*—H⋯*A*	*D*—H	H⋯*A*	*D*⋯*A*	*D*—H⋯*A*
C17—H17*A*⋯O2^i^	0.98	2.44	3.3712 (18)	158

Symmetry code: (i) 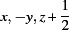.

**Fig. 1.**
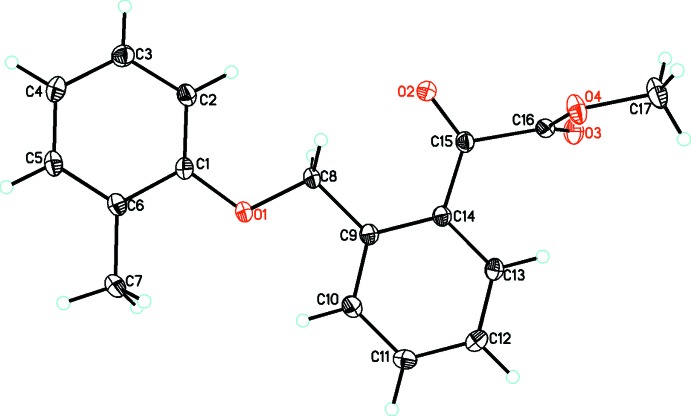
Molecular structure of the title compound showing 30% probability displacement ellipsoids.

**Fig. 2.**
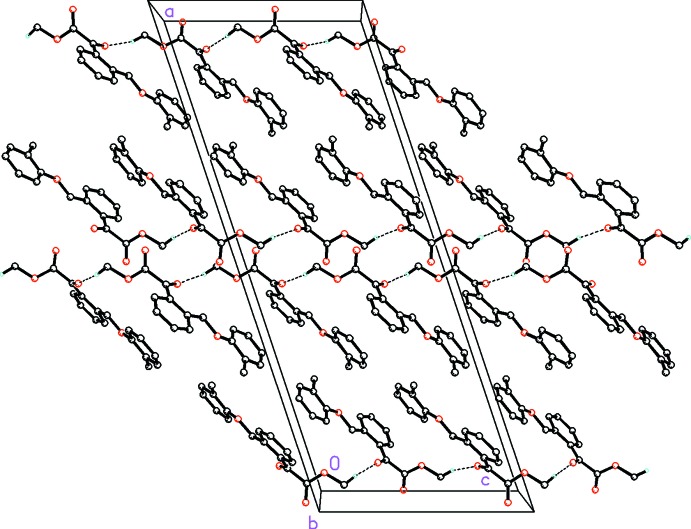
Packing diagram of the title compound viewed along the b axis. Dashed lines indicate weak C—H···O intermolecular interactions. H atoms not involved in these weak intermolecular interactions have been omitted for clarity.

## supplementary crystallographic information

### Comment

The title compound (I) is used in organic synthesis as a fungicide intermediate and mainly used as an intermediate for the preparation of methyl 2(E)-methoxyimino-2-[2-(2-methylphenoxymethyl)phenyl] acetate or kresoxim-methyl, which is an active agrochemical exhibiting fungicidal activity (Ammermann *et al.*, 2000; Balba, 2007; Cash & Cronan, 2001). The crystal structure of kresoxim-methyl (Chopra *et al.*, 2004) and 2-[(E)-methoxyimino]-2-{2-[(2-methylphenoxy) methyl]phenyl}ethanoic acid (Kant *et al.*, 2012) have been reported. In view of the importance of the title compound, this paper reports its crystal structure.

In (I), the dihedral angle between the mean planes of the benzene rings is 4.4 (2)° (Fig. 1). Bond lengths are in normal ranges (Allen *et al.*, 1987). In the crystal, weak C—H···O hydrogen bonds connect (Table 1) molecules along [001] (Fig. 2).

### Experimental

The title compound was a gift sample from RL Fine Chem, Bengaluru, India. The compound was recrystallized from methyl t-butyl ether by slow evaporation (M.P.: 322–323 K).

### Refinement

All H atoms were placed in calculated positions and refined using a riding-model approximation with C—H lengths of 0.95Å (CH), 0.99Å (CH_2_) or 0.98Å (CH_3_). Isotropic displacement parameters for these atoms were set to 1.2 (CH, CH_2_) or 1.5 (CH_3_) times *U*_eq_ of the parent atom. Secondary CH_2_ refined with riding coordinates: C8(H8A,H8B.). Aromatic H refined with riding coordinates: C2(H2), C3(H3), C4(H4), C5(H5), C10(H10), C11(H11), C12(H12), C13(H13). Idealised Me refined as rotating group: C7(H7A,H7B,H7C), C17(H17A,H17B,H17C).

### Crystal data

**Table:**

C_17_H_16_O_4_	*F*(000) = 1200
*M**_r_* = 284.30	*D*_x_ = 1.316 Mg m^−^^3^
Monoclinic, *C*2/*c*	Cu *K*α radiation, λ = 1.54184 Å
*a* = 31.6697 (11) Å	Cell parameters from 3111 reflections
*b* = 7.5883 (2) Å	θ = 2.9–75.3°
*c* = 12.5915 (6) Å	µ = 0.77 mm^−^^1^
β = 108.514 (4)°	*T* = 123 K
*V* = 2869.4 (2) Å^3^	Prism, colorless
*Z* = 8	0.47 × 0.34 × 0.14 mm

### Data collection

**Table:**

Agilent Xcalibur (Ruby, Gemini) diffractometer	2551 reflections with *I* > 2σ(*I*)
Detector resolution: 10.5081 pixels mm^-1^	*R*_int_ = 0.021
ω scans	θ_max_ = 75.5°, θ_min_ = 2.9°
Absorption correction: multi-scan (*CrysAlis RED*; Agilent, 2012)	*h* = −39→35
*T*_min_ = 0.921, *T*_max_ = 1.000	*k* = −8→9
5506 measured reflections	*l* = −12→15
2897 independent reflections	

### Refinement

**Table:**

Refinement on *F*^2^	0 restraints
Least-squares matrix: full	Hydrogen site location: inferred from neighbouring sites
*R*[*F*^2^ > 2σ(*F*^2^)] = 0.038	H-atom parameters constrained
*wR*(*F*^2^) = 0.107	*w* = 1/[σ^2^(*F*_o_^2^) + (0.0554*P*)^2^ + 1.2142*P*], where *P* = (*F*_o_^2^ + 2*F*_c_^2^)/3
*S* = 1.07	(Δ/σ)_max_ = 0.001
2897 reflections	Δρ_max_ = 0.23 e Å^−^^3^
192 parameters	Δρ_min_ = −0.20 e Å^−^^3^

### Special details

**Table:**

Geometry. All esds (except the esd in the dihedral angle between two l.s. planes) are estimated using the full covariance matrix. The cell esds are taken into account individually in the estimation of esds in distances, angles and torsion angles; correlations between esds in cell parameters are only used when they are defined by crystal symmetry. An approximate (isotropic) treatment of cell esds is used for estimating esds involving l.s. planes.

### Fractional atomic coordinates and isotropic or equivalent isotropic displacement parameters (Å^2^)

**Table:**

	*x*	*y*	*z*	*U*_iso_*/*U*_eq_	
O1	0.15989 (3)	0.23976 (12)	0.71615 (8)	0.0269 (2)	
O2	0.05640 (3)	0.09672 (13)	0.83488 (8)	0.0334 (2)	
O3	−0.01120 (3)	0.31730 (14)	0.91715 (8)	0.0328 (2)	
O4	0.04621 (3)	0.20101 (15)	1.05392 (8)	0.0345 (3)	
C1	0.17209 (4)	0.09964 (17)	0.66403 (10)	0.0228 (3)	
C2	0.15067 (4)	−0.06284 (18)	0.64687 (10)	0.0257 (3)	
H2	0.1261	−0.0829	0.6734	0.031*	
C3	0.16520 (4)	−0.19597 (18)	0.59084 (11)	0.0298 (3)	
H3	0.1507	−0.3071	0.5795	0.036*	
C4	0.20081 (5)	−0.16704 (19)	0.55151 (11)	0.0315 (3)	
H4	0.2105	−0.2572	0.5124	0.038*	
C5	0.22218 (4)	−0.00414 (19)	0.57003 (10)	0.0286 (3)	
H5	0.2467	0.0149	0.5434	0.034*	
C6	0.20871 (4)	0.13107 (18)	0.62609 (10)	0.0238 (3)	
C7	0.23140 (4)	0.30732 (19)	0.64563 (12)	0.0306 (3)	
H7A	0.2576	0.3039	0.6200	0.046*	
H7B	0.2408	0.3351	0.7258	0.046*	
H7C	0.2107	0.3979	0.6039	0.046*	
C8	0.12311 (4)	0.21425 (17)	0.75746 (11)	0.0243 (3)	
H8A	0.1293	0.1145	0.8109	0.029*	
H8B	0.0958	0.1870	0.6947	0.029*	
C9	0.11668 (4)	0.38121 (17)	0.81511 (10)	0.0224 (3)	
C10	0.14305 (4)	0.52884 (18)	0.81747 (11)	0.0267 (3)	
H10	0.1648	0.5250	0.7802	0.032*	
C11	0.13820 (4)	0.68171 (18)	0.87322 (11)	0.0287 (3)	
H11	0.1566	0.7806	0.8735	0.034*	
C12	0.10674 (4)	0.69138 (17)	0.92858 (11)	0.0278 (3)	
H12	0.1033	0.7964	0.9662	0.033*	
C13	0.08042 (4)	0.54612 (17)	0.92822 (10)	0.0247 (3)	
H13	0.0591	0.5515	0.9668	0.030*	
C14	0.08456 (4)	0.39122 (17)	0.87205 (10)	0.0222 (3)	
C15	0.05692 (4)	0.23845 (17)	0.87950 (10)	0.0243 (3)	
C16	0.02595 (4)	0.26027 (16)	0.95160 (11)	0.0240 (3)	
C17	0.02123 (5)	0.2110 (2)	1.13287 (13)	0.0395 (4)	
H17A	0.0373	0.1474	1.2015	0.059*	
H17B	−0.0082	0.1577	1.0994	0.059*	
H17C	0.0177	0.3347	1.1509	0.059*	

### Atomic displacement parameters (Å^2^)

**Table:**

	*U*^11^	*U*^22^	*U*^33^	*U*^12^	*U*^13^	*U*^23^
O1	0.0267 (4)	0.0268 (5)	0.0336 (5)	−0.0032 (4)	0.0187 (4)	−0.0044 (4)
O2	0.0380 (5)	0.0261 (5)	0.0447 (6)	−0.0063 (4)	0.0254 (4)	−0.0081 (4)
O3	0.0250 (5)	0.0420 (6)	0.0332 (5)	0.0060 (4)	0.0117 (4)	−0.0003 (4)
O4	0.0296 (5)	0.0466 (6)	0.0335 (5)	0.0129 (4)	0.0189 (4)	0.0124 (4)
C1	0.0212 (5)	0.0276 (6)	0.0205 (5)	0.0017 (5)	0.0080 (4)	−0.0008 (5)
C2	0.0218 (6)	0.0308 (7)	0.0260 (6)	−0.0019 (5)	0.0095 (5)	−0.0017 (5)
C3	0.0296 (7)	0.0288 (7)	0.0302 (7)	−0.0023 (5)	0.0084 (5)	−0.0042 (5)
C4	0.0339 (7)	0.0340 (7)	0.0292 (7)	0.0057 (6)	0.0138 (5)	−0.0046 (6)
C5	0.0265 (6)	0.0366 (7)	0.0269 (6)	0.0047 (5)	0.0143 (5)	0.0027 (5)
C6	0.0211 (5)	0.0299 (6)	0.0211 (5)	0.0011 (5)	0.0077 (4)	0.0028 (5)
C7	0.0281 (6)	0.0338 (7)	0.0345 (7)	−0.0043 (5)	0.0163 (5)	0.0008 (6)
C8	0.0230 (6)	0.0268 (6)	0.0278 (6)	−0.0016 (5)	0.0145 (5)	−0.0017 (5)
C9	0.0218 (5)	0.0251 (6)	0.0204 (5)	0.0013 (5)	0.0068 (4)	0.0010 (5)
C10	0.0251 (6)	0.0293 (7)	0.0277 (6)	−0.0015 (5)	0.0112 (5)	0.0007 (5)
C11	0.0272 (6)	0.0251 (6)	0.0329 (7)	−0.0035 (5)	0.0083 (5)	0.0008 (5)
C12	0.0287 (6)	0.0243 (6)	0.0285 (6)	0.0019 (5)	0.0064 (5)	−0.0041 (5)
C13	0.0226 (6)	0.0275 (6)	0.0247 (6)	0.0032 (5)	0.0086 (4)	−0.0009 (5)
C14	0.0209 (5)	0.0239 (6)	0.0222 (5)	0.0013 (5)	0.0074 (4)	0.0006 (5)
C15	0.0227 (6)	0.0264 (6)	0.0261 (6)	0.0015 (5)	0.0109 (5)	0.0001 (5)
C16	0.0241 (6)	0.0214 (6)	0.0292 (6)	−0.0011 (5)	0.0124 (5)	−0.0006 (5)
C17	0.0421 (8)	0.0485 (9)	0.0376 (8)	0.0142 (7)	0.0265 (6)	0.0154 (7)

### Geometric parameters (Å, º)

**Table:**

O1—C1	1.3681 (15)	C7—H7C	0.9800
O1—C8	1.4315 (13)	C8—H8A	0.9900
O2—C15	1.2111 (16)	C8—H8B	0.9900
O3—C16	1.1981 (16)	C8—C9	1.5058 (17)
O4—C16	1.3224 (16)	C9—C10	1.3919 (18)
O4—C17	1.4560 (15)	C9—C14	1.4204 (16)
C1—C2	1.3907 (18)	C10—H10	0.9500
C1—C6	1.4078 (16)	C10—C11	1.3894 (19)
C2—H2	0.9500	C11—H11	0.9500
C2—C3	1.3915 (18)	C11—C12	1.3876 (18)
C3—H3	0.9500	C12—H12	0.9500
C3—C4	1.3855 (19)	C12—C13	1.3810 (19)
C4—H4	0.9500	C13—H13	0.9500
C4—C5	1.393 (2)	C13—C14	1.3993 (17)
C5—H5	0.9500	C14—C15	1.4736 (17)
C5—C6	1.3871 (18)	C15—C16	1.5423 (16)
C6—C7	1.5010 (18)	C17—H17A	0.9800
C7—H7A	0.9800	C17—H17B	0.9800
C7—H7B	0.9800	C17—H17C	0.9800
			
C1—O1—C8	117.10 (10)	C9—C8—H8B	110.1
C16—O4—C17	116.53 (10)	C10—C9—C8	121.03 (11)
O1—C1—C2	124.43 (11)	C10—C9—C14	117.85 (11)
O1—C1—C6	114.82 (11)	C14—C9—C8	121.09 (11)
C2—C1—C6	120.75 (12)	C9—C10—H10	119.3
C1—C2—H2	120.0	C11—C10—C9	121.40 (12)
C1—C2—C3	119.93 (11)	C11—C10—H10	119.3
C3—C2—H2	120.0	C10—C11—H11	119.7
C2—C3—H3	119.9	C12—C11—C10	120.67 (12)
C4—C3—C2	120.26 (13)	C12—C11—H11	119.7
C4—C3—H3	119.9	C11—C12—H12	120.5
C3—C4—H4	120.4	C13—C12—C11	119.02 (12)
C3—C4—C5	119.21 (12)	C13—C12—H12	120.5
C5—C4—H4	120.4	C12—C13—H13	119.4
C4—C5—H5	119.0	C12—C13—C14	121.25 (11)
C6—C5—C4	122.02 (12)	C14—C13—H13	119.4
C6—C5—H5	119.0	C9—C14—C15	121.69 (11)
C1—C6—C7	119.89 (11)	C13—C14—C9	119.81 (11)
C5—C6—C1	117.83 (12)	C13—C14—C15	118.40 (11)
C5—C6—C7	122.28 (11)	O2—C15—C14	126.15 (11)
C6—C7—H7A	109.5	O2—C15—C16	116.80 (11)
C6—C7—H7B	109.5	C14—C15—C16	117.05 (11)
C6—C7—H7C	109.5	O3—C16—O4	126.33 (12)
H7A—C7—H7B	109.5	O3—C16—C15	124.09 (12)
H7A—C7—H7C	109.5	O4—C16—C15	109.53 (10)
H7B—C7—H7C	109.5	O4—C17—H17A	109.5
O1—C8—H8A	110.1	O4—C17—H17B	109.5
O1—C8—H8B	110.1	O4—C17—H17C	109.5
O1—C8—C9	108.15 (10)	H17A—C17—H17B	109.5
H8A—C8—H8B	108.4	H17A—C17—H17C	109.5
C9—C8—H8A	110.1	H17B—C17—H17C	109.5
			
O1—C1—C2—C3	178.72 (11)	C8—C9—C14—C13	177.75 (11)
O1—C1—C6—C5	−178.46 (11)	C8—C9—C14—C15	1.40 (17)
O1—C1—C6—C7	0.78 (16)	C9—C10—C11—C12	0.1 (2)
O1—C8—C9—C10	2.58 (16)	C9—C14—C15—O2	−4.4 (2)
O1—C8—C9—C14	−175.27 (10)	C9—C14—C15—C16	175.10 (10)
O2—C15—C16—O3	−92.56 (17)	C10—C9—C14—C13	−0.16 (17)
O2—C15—C16—O4	85.23 (15)	C10—C9—C14—C15	−176.51 (11)
C1—O1—C8—C9	177.79 (10)	C10—C11—C12—C13	0.47 (19)
C1—C2—C3—C4	−0.34 (19)	C11—C12—C13—C14	−0.85 (19)
C2—C1—C6—C5	0.85 (18)	C12—C13—C14—C9	0.70 (18)
C2—C1—C6—C7	−179.91 (12)	C12—C13—C14—C15	177.17 (11)
C2—C3—C4—C5	0.8 (2)	C13—C14—C15—O2	179.20 (12)
C3—C4—C5—C6	−0.5 (2)	C13—C14—C15—C16	−1.30 (17)
C4—C5—C6—C1	−0.35 (19)	C14—C9—C10—C11	−0.21 (18)
C4—C5—C6—C7	−179.57 (12)	C14—C15—C16—O3	87.90 (16)
C6—C1—C2—C3	−0.51 (19)	C14—C15—C16—O4	−94.31 (13)
C8—O1—C1—C2	1.38 (17)	C17—O4—C16—O3	−1.9 (2)
C8—O1—C1—C6	−179.34 (10)	C17—O4—C16—C15	−179.59 (12)
C8—C9—C10—C11	−178.12 (11)		

### Hydrogen-bond geometry (Å, º)

**Table:**

*D*—H···*A*	*D*—H	H···*A*	*D*···*A*	*D*—H···*A*
C17—H17*A*···O2^i^	0.98	2.44	3.3712 (18)	158

Symmetry code: (i) *x*, −*y*, *z*+1/2.
